# Effective Detergency Determination for Single Polymeric Fibers Using Confocal Microscopy

**DOI:** 10.3390/polym15153314

**Published:** 2023-08-05

**Authors:** Qian Hu, Jindan Wu, Zhiqiang Qin, Xuanxiang Wei, Chenchen Jiang, Minghua Wu, Deyou Yu, Jiping Wang

**Affiliations:** 1 College of Textile Science and Engineering, Zhejiang Sci-Tech University, Hangzhou 310018, Chinawujindan@zstu.edu.cn (J.W.);; 2 Zheijiang Sci-Tech University Xiangshan Research Institute, Ningbo 315700, China; 3 Hubei Provincial Engineering Laboratory for Clean Production and High Value Utilization of Bio-Based Textile Materials, Wuhan Textile University, Wuhan 430200, China; 4 School of Textiles and Fashion, Shanghai University of Engineering Science, Shanghai 201620, China

**Keywords:** confocal laser scanning microscope, single polymeric fiber, detergency, fluorescence intensity, oil removal

## Abstract

Detergency determination for single polymeric fibers is of significant importance to screening effective detergents for laundry, but remains challenging. Herein, we demonstrate a novel and effective method to quantify the detergency for single polymeric fibers using a confocal laser scanning microscope (CLSM). It was applied to visualize the oil-removing process of single polymeric fibers and thus assess the detergency of various detergents. Four typical surfactants were selected for comparison, and a compounded detergent containing multiple components (e.g., anionic and nonionic surfactants, enzymes) was demonstrated to be the most effective and powerful soil-removing detergent because more than 50% of oil on the cotton fiber could be easily removed. Moreover, the oil removal process of three kinds of fibers (i.e., cotton, viscose, and polyester) was imaged and monitored by confocal microscopy. It was found that the percentage of the detergency of a single polyester fiber exceeded 70%, which is much higher than that of cotton and viscose fibers (~50%), which may be due to its relatively smooth surface. Compared to traditional methods, the CLSM imaging method is more feasible and effective to determine the detergency of detergents for single polymeric fibers.

## 1. Introduction

Detergency is the capability of removing undesired substances from apparel or textiles, which has gained increasing attention in the fabric cleaning process during home laundry [1,2]. It has been found that soil removal is a complex process affected by several factors, such as the properties of substrate, the conditions of the laundry (concentration of the surfactant, hardness of the water, temperature, time, equipment, etc.), the type of soil, and the composition of the detergent [3,4,5,6,7,8,9]. Soil can be classified into three groups: particulate soil (inorganic solid), oily soil (usually organic liquid), and stains (unwanted dyestuffs) [2,10]. Among them, the removal of oily soil is one of the greatest challenges in cleaning [11]. In particular, soybean oil is considered a suitable oily soil sample for detergency studies due to the wide use of it in cooking. It is a mixture of 22–25% oleic acid, 57–64% linoleic acid, and 10–12% palmitic acid [12,13]. Soybean oil is quite difficult to remove because the highly hydrophobic and bulky fatty acids make it poorly solubilized in surfactant micelles [14,15,16].

Recently, numerous analysis methods for determining detergency in fabric have been developed and improved for applications [17,18,19]. Most of them focus on the changes in the soiled fabric before and after laundry procedures, such as the differences in soil mass, color, physical features, and so on. For instance, the amount of oil soil on fabric before and after the washing process can be quantitatively analyzed by a UV/Vis spectrophotometer, radioisotopes, gas chromatography, and the gravity method. On the other hand, the titration method can also be used to quantify the mass of oil soil removed by the laundry in the washing bath [20,21,22,23,24]. Similarly, a colorimetric spectrophotometer is usually used to determine the variation of fabric color by measuring the parameters, such as the reflectance, brightness, and K/S and ΔE of soiled fabric, before and after cleaning [20,25,26,27]. Moreover, the soil, both on the lengthwise and cross section of the stained fabric, could be roughly observed using a scanning electron microscope (SEM) [28,29], and the detergency performance could be assessed accordingly.

The developed soil amount determination methods are capable of quantification, but they often require complex and time-consuming procedures. In contrast, the colorimetric method is facile, but hard to be observed and monitored. In addition, neither of them is visualizable during the soil-removing process. Therefore, it is imperative to establish a visualizable and quantitative method to evaluate the detergency performance of single polymeric fibers. Confocal laser scanning microscope (CLSM) technology is an advanced and optional candidate widely used in cellular and molecular biology analysis [30,31,32,33]. In general, the sample labeled with the fluorescent substance could be excited by laser and emit fluorescence under CLSM observation. The fluorescence intensity is associated with the amount of fluorescent substance, and the latter reflects the amount of sample. In this context, the in-situ amount change of sample is expected to be discovered and monitored by confocal microscopy [34].

Herein, we demonstrate, for the first time, a novel and facile method to image and determine soybean oil removal from different polymeric fibers based on confocal microscopy. Four typical surfactants and three types of fibers (cotton, viscose, and polyester) were selected to assess the soybean oil removal and feasibility of the proposed determination method. Moreover, three common methods (colorimetric method, SEM observing method, and gravity method) were also investigated for comparison. Nile Red, a fluorescent substance, was applied to label the oil soil on the fiber in order to image the detergency process [35]. The per unit area relative fluorescence intensity was calculated to evaluate the oil amount variation and thereby quantify the detergency. Our work provides a simple and useful method for the rapid measurement of the detergency performance of both detergents and fibers.

## 2. Experimental Procedures

### 2.1. Materials

Analytical grade acetone (A. R.), ethanol (A. R.), and dichloromethane (A. R.) were purchased from Zhejiang Sanying Chemical Agent Co., Ltd. (Ningbo, China). Soybean oil (AAAWANA BAAND) and pure milk (Mengniu) were purchased from the local supermarket. Nile Red (95% purity), which is a kind of fluorescent dye that can be used in the dyeing of soybean oil, was purchased from Aladdin Co., Ltd. (Shanghai, China). Fluorescein isothiocyanate isomer (FITC), which is a kind of fluorescent dye that can be applied for the marking of protein, was purchased from Aladdin Co., Ltd. Sodium dodecyl benzene sulfonate (LAS) and primary alcohol ethoxylate (AEO-9, technical grade with an alkyl chain length average of 12–14) were purchased from Aladdin Co., Ltd. and Linyi Lusen Chemical Co., Ltd. (Linyi, China), respectively. A commercial detergent (Tide Free Liquid Laundry Detergent) was kindly provided by P&G Co., Ltd. (Cincinnati, OH, USA). All chemicals were used as received without further purification. The plain-woven fabrics used in this study were standard unsoiled pure cotton, viscose, and polyester, which were obtained from Shaoxing Furun Dyeing & Finishing Co., Ltd. (Shaoxing, China).

### 2.2. Detergency Determination Procedures for Single Polymeric Fibers

#### 2.2.1. Preparation of Oil-Stained Fibers

The fabrics were washed with water before use in order to eliminate the residues of after-finishing agents. First of all, the yarn samples were first extracted from the pre-washed cotton, viscose, and polyester fabrics. Then, the yarns were completely immerged into the soybean oil for 5 min, and the redundant oil on the yarns was blotted up using filter paper. Subsequently, 0.05 g of Nile Red dye was dissolved in acetone and diluted with distilled water to 50 mL before being applied to the stained yarns. The yarns were immerged into the Nile Red solution for 5 min, and the redundant solution on the yarns was removed using filter paper [36]. At last, the prepared yarns were dried at room temperature for 1 h. The cotton, viscose, and polyester fibers were carefully extracted from the treated yarns for further tests.

#### 2.2.2. Preparation of Protein-Stained Fibers

The preparation of protein-stained fibers was similar to oil-stained fibers, except for following steps: (i) the milk immerged time was set at 1 min; (ii) the protein-stained fibers was soaked in FITC ethanol solution for 2 h dyeing in the refrigerator. 

#### 2.2.3. Washing Procedure

**Figure 1:**
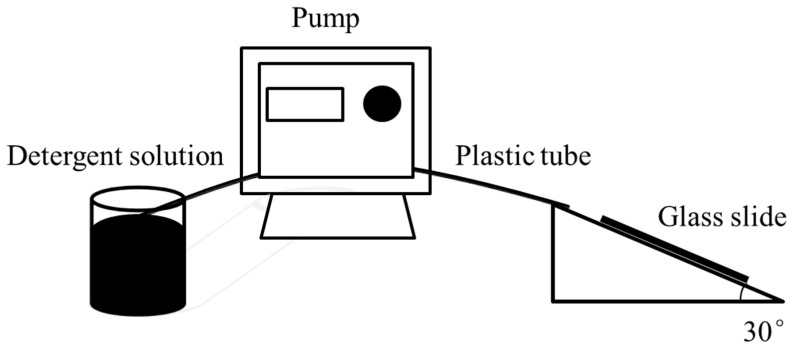
Schematical illustration of the washing process for single polymeric fibers.

The marked fibers were fixed on a glass slide and washed by the flow of detergent solutions at a speed of 36 mL min^−1^ to mimic the typical washing process. A commercial Tide Free liquid detergent, an anionic surfactant (LAS, 15%), a nonionic surfactant (AEO-9, 15%), and a surfactant composition of 15% LAS/AEO-9 (1:1 wt%) were used as detergent formulas. Water with a hardness of 250 ppm (Ca^2+^/Mg^2+^ = 2:1) was prepared and used as the control sample. The washing experiments were carried out at 25 °C with a water hardness of 250 ppm (Ca^2+^/Mg^2+^ = 2:1) and 1% of detergent concentration. Commonly, a detergent concentration of about 0.1% was applied in the daily laundry process. However, considering that a heavier stain was obtained on the fabric and fiber samples than daily textiles according to the oil soaking procedure in experiments, the concentration of detergents increased to 1%. Figure 1 illustrates the simplified schematical diagram of this procedure. In this case, a pump (LongerPump BT100-1F, Baoding, China) was used to control the speed of the detergent flow, and the glass slide, where the fiber was placed, was fixed on a slanted stage. 

#### 2.2.4. In-Situ Monitoring Oil and Protein Removal on Single Polymeric Fibers

The confocal laser scanning microscope (CLSM) (Nikon Eclipse CI, Tokyo, Japan) was used to image the soybean oil and pure milk removal process from single polymeric fibers. The excitation and emission wavelengths were set at 543.5 nm and 650 nm for Nile Red [37], and 488 nm and 543.5 nm for FITC, respectively. The magnification of single cotton and viscose fibers was 400×, while that of single polyester fiber was 200×. The as-prepared single polymeric fibers before and after washing were observed by CLSM, and the fluorescence images were recorded after every 30 s of washing.

In addition, the per unit area relative fluorescence intensity (RFI) based on the obtained CLSM images of the stained fibers before (*I_B_*) and after washing (*I_A_*) was calculated using Image-Pro Plus. The percentage of detergency was calculated by Equation (1):

(1) $${\;\mathrm{Detergency}}_{CLSM}\;\left(\%\right)=\frac{I_{B}-I_{A}}{I_{B}}\times 100\%$$

### 2.3. Detergency Determination Procedures for Fabrics

#### 2.3.1. Soiling Procedure

For comparison, three common methods including the colorimetric method, SEM observing method, and gravity method were used to measure detergency performance. The pre-washed cotton fabric was cut into 2 × 4 inch swatches in the warp and weft direction. The rough selvedge was ripped out in order to avoid the weight loss of the fabric caused by mechanical friction during the laundry procedure. Then, the swatches were placed in a constant temperature humidity chamber overnight prior to use, and 10 mL of soybean oil was added to 90 mL of dichloromethane to afford an oil solution. Each cotton swatch was folded and completely immerged in the oil solution for 5 min. Afterwards, the sample was unfolded and laid on filter paper in order to blot up the redundant oil. Finally, the fabric swatches were dried in an oven and balanced in a temperature humidity chamber overnight prior to use. The average oil content of the soiled fabric was measured to be 15.17 ± 2.68%.

#### 2.3.2. Laundry Procedure

A washing tester (LABTEC HB12P, Shanghai, China) was used to wash the as-prepared cotton fabric swatches. The laundry experiments were performed in washing solution with a liquor ratio of 1:30 (fabric to water) for 20 min. The swathes were then rinsed twice with deionized water for 2 min. All washing experiments were conducted at a washing temperature of 25 °C and a water hardness of 250 ppm (Ca^2+^/Mg^2+^ = 2:1). The detergent formulas were the same as those used in the microscopy study. All experiments were repeated at least three times and the averaged values were assessed for detergency performance evaluation.

#### 2.3.3. Detergency Performance Evaluation

The changes in brightness, reflectance, and weight before and after laundry were calculated. Brightness and reflectance were measured by a color photometer (Datacolor 600SF, Lawrenceville, NJ, USA). The L and R values provided by this instrument are representative of the brightness and reflectance of the fabric. The detergency and oil removal efficiency can be calculated by Equations (2)–(4):

(2) $${\;\mathrm{Detergency}}_{L}=L_{A}-L_{B}$$

(3) $${\mathrm{Detergency}}_{R}=R_{A}-R_{B}$$

(4) $${\mathrm{Detergency}}_{W}=\frac{G_{B}-G_{A}}{G_{B}-G_{C}}\times 100\%\;$$

where *L_B_*, *R**_B_***, and *G**_B_*** are the brightness, reflectance, and weight value of the soiled swatches before laundry, while *L_A_*, *R_A_*, and *G_A_* are the brightness, reflectance, and weight value of the soiled swatches after laundry. *G_C_* is the weight of the unsoiled swatches. The weights of the swatches were measured after being balanced in the constant temperature humidity chamber overnight.

## 3. Results and Discussion

### 3.1. Detergency of Different Detergents

**Figure 2:**
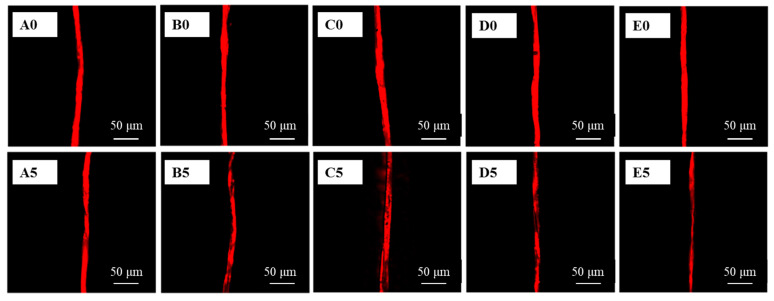
CLSM Images of cotton fibers before and after washing. (**A**): water; (**B**): AEO-9; (**C**): LAS; (**D**): LAS/AEO-9 (1:1 wt%); and (**E**): Tide Free. 0: unwashed; 5: washing for 150 s.

The removal of soybean oil from a single cotton fiber by different detergents was easily imaged by confocal microscopy, and the captured images are shown in Figure 2. The recorded images show that the unwashed stained cotton fibers were covered with soybean oil marked by Nile Red dye. Since the fluorescence intensity of the fibers decreased when the Nile Red-labeled soybean oil was washed away by detergent solution, the variation of RFI could reflect the change in oil amount on the fibers, and therefore could be used as an indicator of detergency. As shown in Figure 2, the RFI of stained cotton fibers before washing showed distinct changes with and without detergency usage. On the one hand, an obvious decrease in RFI was found on fibers after being washed for 150 s with different detergents, including a commercial liquid detergent (Tide Free), nonionic surfactant (AEO-9), anionic surfactant (LAS), and formulated surfactant (LAS/AEO-9). On the other hand, an insignificant change in RFI was found after being rinsed by water, suggesting that the oil removal efficiency of water is relatively weak. Therefore, the oil removal efficiency of different detergents could be quickly prejudged by CLSM images.

**Figure 3:**
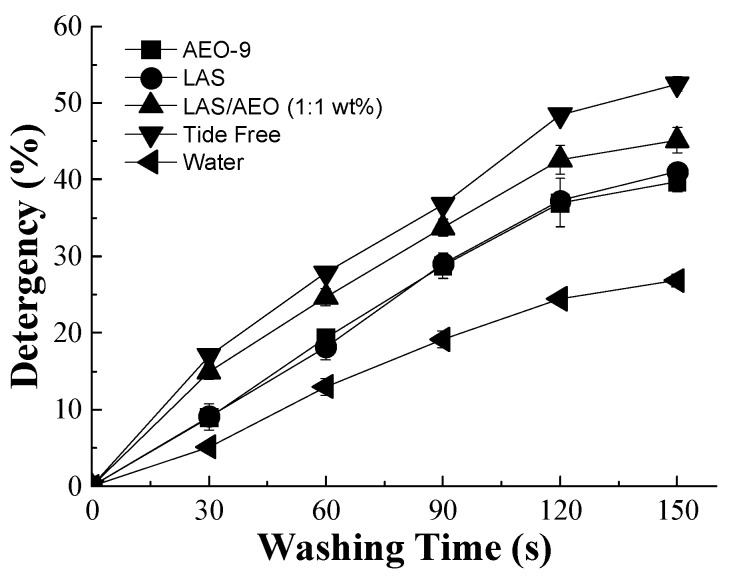
Detergency of single cotton fibers with different detergents (AEO-9, LAS, LAS/AEO-9(1:1), and Tide Free) compared to the water.

To quantify the oil removal efficiency of different detergents, the percentage of RFI variation was calculated using Image-Pro Plus and plotted against the washing time, as depicted in Figure 3. It was shown that the percentage of detergency was in the order of Tide Free > LAS/AEO-9 (1:1 wt%) > LAS > AEO-9 > water. The percentage of the detergency of LAS and AEO-9 was calculated to be 39.7% and 41.0% in 150 s, respectively, while water could only remove 25% of oil. Compared to a single surfactant solution, the compounded solution of LAS and AEO-9 exhibited a higher percentage of detergency (~44%). In the latter case, the solubilization was significantly increased due to the decreased surface tension in the compounded system [38,39]. Similarly, the commercial detergent (Tide Free) containing multiple components (anionic and nonionic surfactants, enzymes) presented the highest oil removal efficiency, since the fluorescent intensity decreased to as low as 48% after washing for 150 s. This result highlights the feasibility and practicality of the proposed CLSM method for the detergency determination of a single polymeric fiber.

### 3.2. Oil Removal Performances of Different Types of Fibers

**Figure 4:**
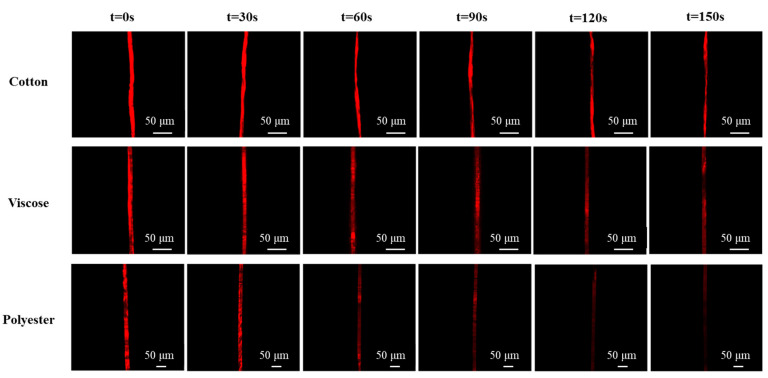
CLSM Images of oil removal from cotton, viscose, and polyester fibers with different washing time by the commercial detergent (Tide Free).

The oil-removing processes of cotton, viscose, and polyester fibers could be easily visualized by CLSM, and the recorded images are shown in Figure 4. The RFI of a single fiber suffer an evident decrease as the washing time increased. Moreover, it can be seen that different types of a single fiber demonstrated various diameters and shapes. The diameters are in the order of polyester (25.87 µm) > cotton (12.40 µm) > viscose (11.37 µm). The cotton fibers are twisted and wrinkled while the viscose and polyester fibers are straight and cylindrical. The shape type of a single fiber had a significant effect on the oil distribution on fibers. The largest amount of oil deposited on cotton fiber was observed while the polyester fiber exhibited the lowest oil uptake. This result may be due to their different surface morphologies and properties. In other words, the wrinkled surface of cotton fiber is favorable for the oil capture in the grooves. In contrast, the oil is hard to be trapped and stored on the smooth surfaces of viscose and polyester fibers.

Distinct differences in the oil removal performance of three types of fibers were also observed. Specifically, much oil remained within the cotton fiber after washing for 90 s (Figure 4). The oil was gradually removed from the viscose fiber, and some parts of oil were unaffected by the detergent after washing for 120 s. The RFI of the soiled polyester fiber decreased fast, and most of the oil was removed at the end of the treatment with the commercial detergent, indicating that the commercial detergent exhibited a fiber-dependent detergency property. This observation would call for future investigations on the development of an effective detergent for targeting fibers.

**Figure 5:**
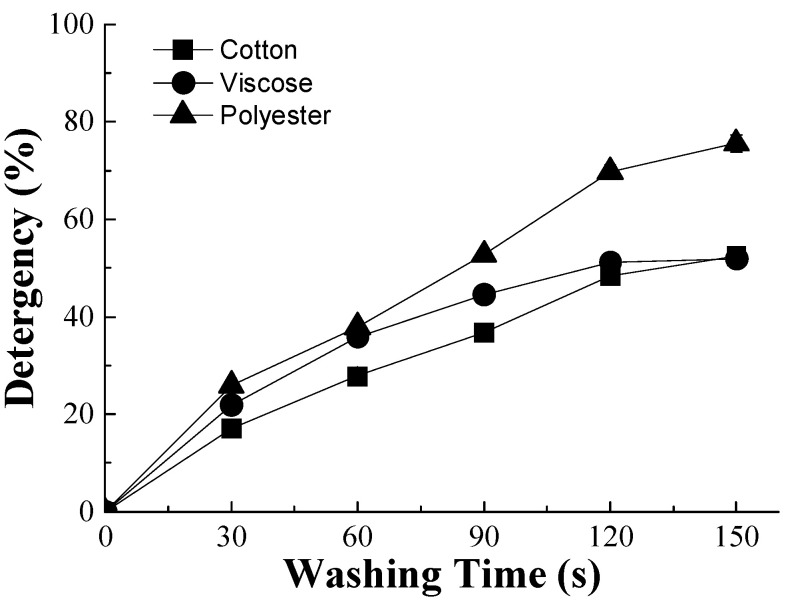
Detergency (RFI) values of cotton, viscose, and polyester fibers with different washing times by the commercial detergent (Tide Free).

In order to compare the oil removal behavior of different single fibers, the percentage of the detergency of the commercial detergent in single cotton, viscose, and polyester fibers was evaluated based on RFI calculation (Figure 5). It was found that for cotton and viscose fibers, the oil removal efficiency increased from 0 to ca. 52%, with a gradual decrease in the increasing rate. About 52% of oil on the cotton was washed away, and the value was close to that of a single viscose fiber, which may be due to their similar chemical components. However, the removal of oil from the viscose fiber is faster than from the cotton fiber at the first washing stage (0~120 s), perhaps because of the cylindrical shape of the viscose fiber. The removal of soybean oil from a single polyester fiber had the fastest rate and the highest amount (ca. 75%,) which may be due to its relatively smooth surface that shows a low affinity to oil.

### 3.3. Comparison of the CLSM Method with Other Common Methods

In order to validate the CLSM imaging method, the detergency performances of different detergents were studied and compared with common measurements. Generally, the parameters, such as brightness (L), reflectance (R), and weight, are applied frequently to evaluate detergency performance at a lab scale. The value of L is usually reported in the range between 0 (black) and 100 (white). A decrease in L indicates an increment in darkness and vice versa [40]. The higher the value of R is, the higher the whiteness of the fabric [41]. Additionally, it was found that the fabric weight loss caused by mechanical friction during the laundry process was less than 0.35%. Thus, the detergency performance on fabric evaluated by the gravity method could deliver valid results. In this context, the value changes in brightness and reflectance reflect the practical color change of the cotton fabric after washing, while the changes in weight and fluorescence intensity reflect the percentage of oil removed by the laundry procedure.

**Figure 6:**
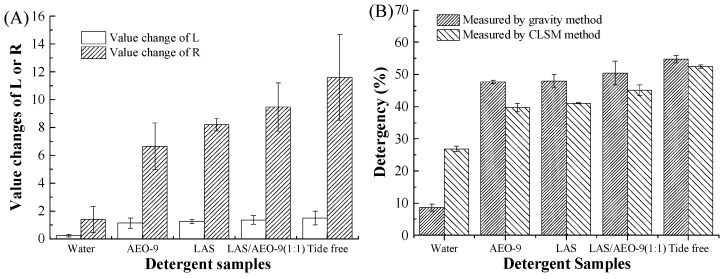
Detergency of detergents in single cotton fiber using different measurements. (**A**) Value changes of L and R; (**B**) detergency measured by gravity method and CLSM imaging method.

Different results were obtained using different measurements (Figure 6). It should be noted that the values of brightness, reflectance, and weight were obtained from cotton fabric, while RFI was obtained from a single cotton fiber. In the case of fabric, the oil removal efficiency is largely affected by the mechanical forces and friction between the fibers and fabrics, which do not occur during the washing procedure of a single fiber. The oil removal efficiency of the commercial detergent measured by the CLSM imaging method based on a single cotton fiber was only influenced by the emulsification effect of detergent. In this case, the CLSM method can truly reflect the detergency performance of detergent. Since the brightness and reflectance parameters were obtained at the same wavelength, their value change was consistent with each other. More importantly, the results obtained by the four methods showed a similar trend with the following detergency order: Tide Free > LAS/AEO-9 (1:1 wt%) > LAS > AEO-9 > water.

**Figure 7:**
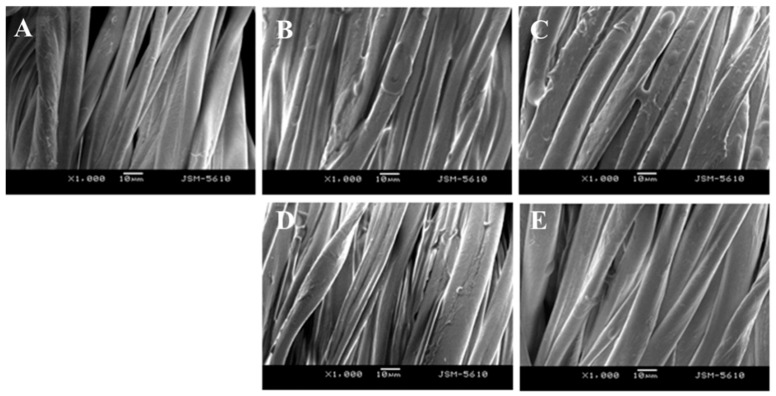
SEM images of cotton fabrics. (**A**): Unsoiled area; (**B**,**C**): soiled area before laundry; (**D**,**E**): soiled area after laundry with water; (**D**): and the commercial detergent (Tide Free) (**E**).

The morphologies of the cotton fabric before and after laundry are displayed in Figure 7. It can be seen that the fiber in the soiled swatches before laundry was completely covered by soybean oil. The oil was observed to occupy the interspace between fiber and fiber in the fabrics, while the oil only covered one single fiber in the developed CLSM method. It was much easier to wash the oil on a single fiber since the oil was not trapped by the interspace of fibers. Therefore, it is not surprising that the detergency of water measured by CLSM was much higher than that measured by the gravity method. Overall, the CLSM method is more sensitive than the other three ones in terms of visualization and quantification, suggesting its high feasibility of detergency determination for single polymeric fibers.

### 3.4. The Applicability of the CLSM Method to Examine Protein Stains

**Figure 8:**
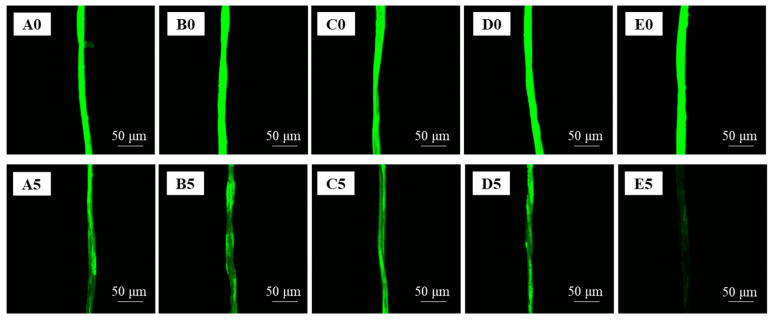
CLSM Images of cotton fibers stained by pure milk before and after washing. (**A**): water; (**B**): AEO-9; (**C**): LAS; (**D**): LAS/AEO-9 (1:1 wt%); and (**E**): Tide Free. 0: unwashed; 5: washing for 150 s.

The CLSM method was applied for examining protein stains to explore whether the novel method could be used in other types of stains. The removal of pure milk from a single cotton fiber by water and different detergents was imaged by confocal microscopy, and the recorded images are shown in Figure 8. The recorded images showed the green fluorescence of FITC, which was responsible for the bind with pure milk. Since the fluorescence intensity of the fibers decreased when the FITC marked pure milk was removed by the detergent process, the variation of RFI could reflect the change in the pure milk amount on the fibers. Therefore, it could be used as a useful indicator of the detergency for protein stains. As shown in Figure 8, the RFI of stained cotton fibers showed an obvious decrease after being washed for 150 s, and a distinct change was observed among different detergents, including Tide Free, AEO-9, LAS, and LAS/AEO-9.

**Figure 9:**
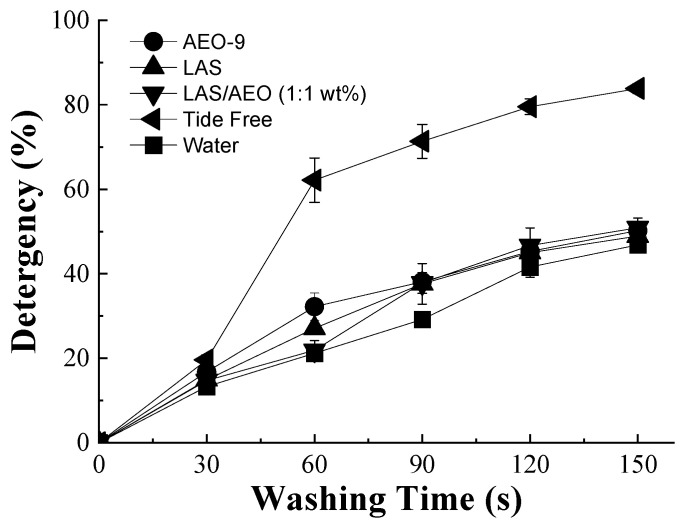
Detergency of single cotton fibers with water and different detergents (AEO-9, LAS, LAS/AEO-9(1:1), and Tide Free).

The pure milk removal efficiency for water and different detergents based on RFI was quantified, and the results are depicted in Figure 9. It was shown that the percentage of detergency was in the order of Tide Free > LAS/AEO-9 (1:1 wt%) > LAS > AEO-9 > water, which was consistent with the order of oil removal efficiency. Moreover, the performance of the pure milk removal of water and other four types of detergents was better than that of oil removal, which is due to the hydrophilic nature of pure milk. This result highlights the applicability of the CLSM method to protein stains and shows the great potential in extending this application to various types of stains.

## 4. Conclusions

In summary, we demonstrate a novel and effective method to quantify the detergency for single polymeric fibers using a confocal laser scanning microscope (CLSM). The oil-removing process of single polymeric fibers could be visualized, and the detergency of various detergents was facilely determined by the change in relative fluorescence intensity. In comparison, a compounded detergent (i.e., Tide Free) containing multiple components exhibited the most effective and powerful soil-removing detergency because more than 50% of oil could be easily removed from a single cotton fiber. The percentage of the detergency of these four surfactants was in the order of Tide Free > LAS/AEO-9 (1:1 wt%) > LAS > AEO-9. In addition, the oil removal process was easily imaged and monitored by confocal microscopy, which provided a fast pre-evaluation of the detergency. It was found that the percentage of the detergency of a single polyester fiber exceeded 70%, which was much higher than that of cotton and viscose fibers (~50%), which may be due to its relatively smooth surface. Compared to traditional methods, the CLSM imaging method is more feasible and effective to determine the detergency of detergents. This work paves the way for the fast and facile determination of the detergency for single polymeric fibers.

## Data Availability

The data are available from the corresponding authors upon reasonable request.

## References

[1] Salahuddin M., Lee Y.-A. (2022). Are Laundry Balls a Sustainable Washing Option for Consumers? Investigating the Effect of Laundry Balls on Microfiber Pollution through the Lens of Cradle-to-Cradle Design Model. Sustainability.

[2] Kalak T., Knast P., Piepiórka-Stepuk J. (2021). The influence of the addition of anionic sodium bis-(2-ethylhexyl) sulfosuccinate on washing properties of liquid laundry detergents. J. Text. Inst..

[3] Verma S., Kumar V.V. (1998). Relationship between Oil–Water Interfacial Tension and Oily Soil Removal in Mixed Surfactant Systems. J. Colloid Interface Sci..

[4] Whang H.S., Kim Y.-J., Ko S.-W., Cha O.S. (2001). Effect of Hydrophile-Lipophile Balance Values of Surfactant Mixtures on the Detergency of Oil-Soiled Single Fibers. Text. Res. J..

[5] Raney K.H., Benson H.L. (1990). The effect of polar soil components on the phase inversion temperature and optimum detergency conditions. J. Am. Oil Chem. Soc..

[6] Park C.H., Chung S.E., Yun C.S. (2007). Effect of drying condition on the electrostatic characteristics of the laundry. Fibers Polym..

[7] Paul B.K., Moulik S.P. (2001). Uses and applications of microemulsions. Curr. Sci..

[8] Chanwattanakit J., Scamehorn J.F., Sabatini D.A., Chavadej S. (2019). Laundry Detergency of Solid Nonparticulate Soil or Waxy Solids: Part II. Effect of the Surfactant Type. J. Surfactants Deterg..

[9] Han H.R., Chung S.E., Kim J., Park C.H. (2015). Mechanical and physicochemical contribution in removal of different soil types on cotton fabric. Text. Res. J..

[10] Carroll B. (1996). The direct study of oily soil removal from solid substrates in detergency. Colloids Surf. A Physicochem. Eng. Asp..

[11] Chanwattanakit J., Scamehorn J.F., Sabatini D.A., Chavadej S. (2017). Laundry Detergency of Solid Non-particulate Soil or Waxy Solids: Part I. Relation to Oily Soil Removal Above the Melting Point. J. Surfactants Deterg..

[12] Song H., Taylor D.C., Zhang M. (2023). Bioengineering of Soybean Oil and Its Impact on Agronomic Traits. Int. J. Mol. Sci..

[13] Heggs R.P., Pierce B.C., Balbes L.M., McRoberts K.C., Streicker M.A., Cockerline K.B. (2020). Assessing Rodent Gnawing of Elastomers Containing Soybean Oil Derivatives. ACS Sustain. Chem. Eng..

[14] Clemente T.E., Cahoon E.B. (2009). Soybean Oil: Genetic Approaches for Modification of Functionality and Total Content. Plant Physiol..

[15] Schwab A.W., Dykstra G.J., Selke E., Sorenson S.C., Pryde E.H. (1988). Diesel fuel from thermal decomposition of soybean oil. J. Am. Oil Chem. Soc..

[16] Timms R.E. (1985). Physical properties of oils and mixtures of oils. J. Am. Oil Chem. Soc..

[17] Gotoh K. (2017). Experimental Analysis of Detergency Phenomena and Investigation of a Next-generation Detergency System. J. Oleo Sci..

[18] Ilec E., Simončič B., Hladnik A. (2009). Evaluation of Surfactant Detergency using Statistical Analysis. Text. Res. J..

[19] Oya M. (2018). Relation between mechanism of soil removal from fabrics and a parameter derived from probability density functional method for washing force analysis. Text. Res. J..

[20] Goel S.K. (1998). Measuring detergency of oily soils in the vicinity of phase inversion temperatures of commercial nonionic surfactants using an oil-soluble dye. J. Surfactants Deterg..

[21] Breen N.E., Durnam D.J., Obendorf S.K. (1984). Residual Oily Soil Distribution on Polyester/Cotton Fabric After, Laundering with Selected Detergents at Various Wash Temperatures. Text. Res. J..

[22] Jurado E., Bravo V., Núñez-Olea J., Bailón R., Altmajer-Vaz D., Garíia-Román M., Fernández-Arteaga A. (2006). Enzyme-based detergent formulas for fatty soils and hard surfaces in a continuous-flow device. J. Surfactants Deterg..

[23] Fujii T., Tatara T., Minagawa M. (1986). Studies on applications of lipolytic enzyme in detergency I. Effect of lipase fromCandida cylindracea on removal of olive oil from cotton fabric. J. Am. Oil Chem. Soc..

[24] Do L.D., Attaphong C., Scamehorn J.F., Sabatini D.A. (2015). Detergency of Vegetable Oils and Semi-Solid Fats Using Microemulsion Mixtures of Anionic Extended Surfactants: The HLD Concept and Cold Water Applications. J. Surfactants Deterg..

[25] Pukale D.D., Bansode A.S., Pinjari D.V., Kulkarni R.R., Sayed U. (2017). Application of Silicone Surfactant Along with Hydrocarbon Surfactants to Textile Washing for the Removal of Different Complex Stains. J. Surfactants Deterg..

[26] Kalak T., Gąsior K., Wieczorek D., Cierpiszewski R. (2020). Improvement of washing properties of liquid laundry detergents by modification with N-hexadecyl-N,N-dimethyl-3-ammonio-1-propanesulfonate sulfobetaine. Text. Res. J..

[27] Gotoh K., Harayama K. (2013). Application of ultrasound to textiles washing in aqueous solutions. Ultrason. Sonochemistry.

[28] Webb J.J., Obendorf S.K. (1988). Detergency study of the synergism between oily and particulate soil on polyester/cotton fabric. J. Am. Oil Chem. Soc..

[29] Chi Y.-S., Obendorf S.K. (1999). Detergency of used motor oil from cotton and polyester fabrics. J. Surfactants Deterg..

[30] Stachelek P., MacKenzie L., Parker D., Pal R. (2022). Circularly polarised luminescence laser scanning confocal microscopy to study live cell chiral molecular interactions. Nat. Commun..

[31] Kim T., Zhou R., Mir M., Babacan S.D., Carney P.S., Goddard L.L., Popescu G. (2014). White-light diffraction tomography of unlabelled live cells. Nat. Photonics.

[32] Mehta V.N., Jha S., Basu H., Singhal R.K., Kailasa S.K. (2015). One-step hydrothermal approach to fabricate carbon dots from apple juice for imaging of mycobacterium and fungal cells. Sens. Actuators B Chem..

[33] Alam A.-M., Park B.-Y., Ghouri Z.K., Park M., Kim H.-Y. (2015). Synthesis of carbon quantum dots from cabbage with down- and up-conversion photoluminescence properties: Excellent imaging agent for biomedical applications. Green Chem..

[34] Greenspan P., Fowler S.D. (1985). Spectrofluorometric studies of the lipid probe, nile red. J. Lipid Res..

[35] Schmid M., Thill A., Purkhold U., Walcher M., Bottero J.Y., Ginestet P., Nielsen P.H., Wuertz S., Wagner M. (2003). Characterization of activated sludge flocs by confocal laser scanning microscopy and image analysis. Water Res..

[36] Pei L., Ge H., Wang D., Zhong Q., Wang J. (2014). The Influence of Silicone Softeners on Fabric Stain Removal and Whiteness Maintenance During Home Laundry. J. Surfactants Deterg..

[37] Cole T.A., Fok A.K., Ueno M.S., Allen R.D. (1990). Use of nile red as a rapid measure of lipid content in ciliates. Eur. J. Protistol..

[38] Wentworth C.M., Castonguay A.C., Moerman P.G., Meredith C.H., Balaj R.V., Cheon S.I., Zarzar L.D. (2022). Chemically Tuning Attractive and Repulsive Interactions between Solubilizing Oil Droplets. Angew. Chem. Int. Ed..

[39] Dong Y., Jin Y., Wei D. (2007). Surface activity and solubilization of a novel series of functional polyurethane surfactants. Polym. Int..

[40] Hutchings J.B., Hutchings J.B. (1999). Food Colour and Appearance in Perspective. Food Colour and Appearance.

[41] Schott H. (1975). A kinetic study of fabric detergency. J. Am. Oil Chem. Soc..

